# Assessing right atrial size in patients with tricuspid regurgitation: importance of the right ventricular-focused view

**DOI:** 10.1093/ehjci/jeae186

**Published:** 2024-07-25

**Authors:** Mara Gavazzoni, Luigi P Badano, Giordano Maria Pugliesi, Marco Penso, Diana-Ruxandra Hădăreanu, Pellegrino Ciampi, Samantha Fisicaro, Giorgio Oliverio, Francesca Heilbron, Michele Tomaselli, Denisa Muraru

**Affiliations:** Department of Cardiology, Istituto Auxologico Italiano, IRCCS, Piazzale Brescia 20, 20149 Milan, Italy; Department of Cardiology, Istituto Auxologico Italiano, IRCCS, Piazzale Brescia 20, 20149 Milan, Italy; Department of Medicine and Surgery, University of Milano Bicocca, Piazzale Brescia 20, 20149 Milan, Italy; Department of Medicine and Surgery, University of Milano Bicocca, Piazzale Brescia 20, 20149 Milan, Italy; Department of Cardiology, Istituto Auxologico Italiano, IRCCS, Piazzale Brescia 20, 20149 Milan, Italy; Department of Cardiology, Clinical Emergency County Hospital of Craiova, Craiova, Romania; Catholic University of the Sacred Heart—Fondazione Policlinico Universitario A. Gemelli, IRCCS, Rome, Italy; Department of Cardiology, Istituto Auxologico Italiano, IRCCS, Piazzale Brescia 20, 20149 Milan, Italy; Department of Cardiology, Istituto Auxologico Italiano, IRCCS, Piazzale Brescia 20, 20149 Milan, Italy; Department of Cardiology, Istituto Auxologico Italiano, IRCCS, Piazzale Brescia 20, 20149 Milan, Italy; Department of Cardiology, Istituto Auxologico Italiano, IRCCS, Piazzale Brescia 20, 20149 Milan, Italy; Department of Cardiology, Istituto Auxologico Italiano, IRCCS, Piazzale Brescia 20, 20149 Milan, Italy; Department of Medicine and Surgery, University of Milano Bicocca, Piazzale Brescia 20, 20149 Milan, Italy

**Keywords:** right atrial volume, two-dimensional echocardiography, three-dimensional echocardiography, clinical outcomes, secondary tricuspid regurgitation

## Abstract

**Aims:**

To assess the accuracy of measuring the right atrial volume (RAV) using two-dimensional echocardiography (2DE) in a right ventricular focused (RVF) view compared to the conventional apical four-chamber (4Ch) view in patients with secondary tricuspid regurgitation (STR). We also compared the clinical correlates of the measures obtained using different methods.

**Methods and results:**

The accuracy of RAV measurements obtained between 2DE-4Ch and RVF views in 384 patients with STR were compared using three-dimensional echocardiography (3DE) as a reference. We used the analysis of variance to test the differences among RAVs obtained from the different 2DE and 3DE acquisitions and the receiving operating characteristics (ROC) curves to evaluate the association with the composite endpoint of hospitalization for heart failure or death. Compared to 3DE, RAV was significantly more underestimated when measurements were obtained from 4Ch rather than RVF (−24 vs. −14%, respectively, *P* < 0.001 for both). RAV underestimation in 4Ch and RVF view was relatively larger in lower grades of STR (−28 vs. −17% in mild, −23 vs. −14% in moderate, and −19 vs. −11% in severe STR, *P* = 0.001), and in the atrial compared to ventricular (−28 vs. −22%; *P* = 0.002) STR. RAV measured by 3DE and RVF showed the highest area under the curve (AUC = 0.67 for 3DE vs. 0.64 for RVF, *P* = 0.05), while 4Ch was significantly less related to the outcomes (AUC: 0.61, *P* = 0.021 vs. 3DE RAV).

**Conclusion:**

In patients with STR, the use of RVF view improved the accuracy of 2DE RAV measurement as compared to the conventional 4Ch-derived measurements.

## Graphical Abstract

**Graphical Abstract:**
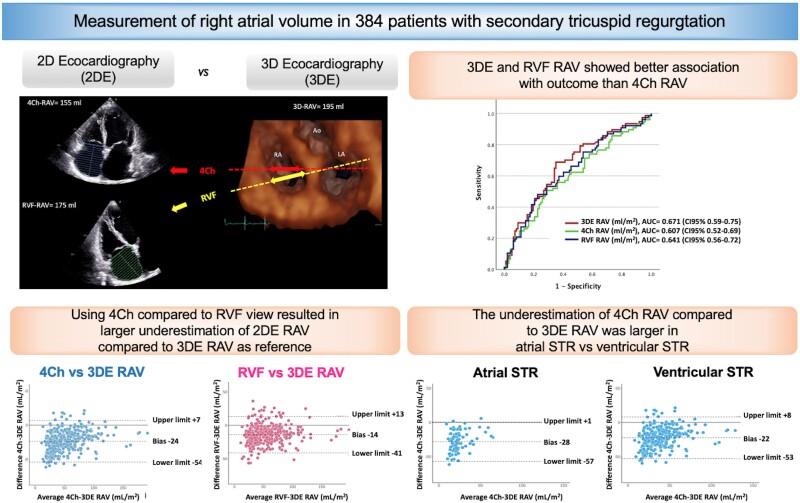
In a population of patients with secondary tricuspid regurgitation (STR), considering three-dimensional echocardiography (3DE) as a reference, the use of a right ventricular focused (RVF) view to measure the right atrial volume (RAV) is compared with the conventional apical four-chamber (4Ch) view; the correlation with clinical outcomes is different among the three methods; the use of RVF significantly reduces the underestimation of RAV; this underestimation is higher in atrial phenotype of STR than in ventricular. 2DE, two-dimensional echocardiography; 3DE, three-dimensional echocardiography; 4Ch, four chamber; Ao, aortic valve; LA, left atrium; RA, right atrium; RAV, right atrial volume; RVF, right ventricular focused; STR, secondary tricuspid regurgitation.

## Introduction

Right atrial volume (RAV) and function are significant predictors of adverse outcomes in different cardiac conditions,^1–13^ including secondary tricuspid regurgitation (STR).^13–18^ In patients with STR, the accurate assessment of RAV has assumed an important role.^13–20^ The dilation of the RA and tricuspid annulus (TA), even in the absence of other pre-disposing conditions, can lead to a specific STR phenotype (atrial STR)^14,16,17,19–21^ that should be differentiated from the ventricular STR because of its different management,^22^ and prognosis.^23–26^

Current guidelines for cardiac chamber quantitation recommend the assessment of RAV at end-systole using the two-dimensional echocardiography (2DE) single-plane area-length or discs’ summation algorithms on an apical four-chamber (4Ch) view focused on RA to avoid its foreshortening.^27^ Although 2DE is the most widely used technique to assess RA dimension, the RAV calculated from the 4Ch has been reported to underestimate the actual RAV measured by either 3DE or cardiac magnetic resonance.^28,29^ The current usage of 3DE for evaluating RAV in clinical practice is uncommon.^30^ This is primarily due to limited evidence demonstrating the superior accuracy of 3DE,^27–29^ recent reporting of reference values for 3DE RAV^31^ and the absence of dedicated software packages for measuring RAV.^29,32^ In order to reduce the underestimation of RAV calculated using 2D 4Ch in routine clinical practice, Ciampi *et al*. have suggested using the 2D echocardiography apical right ventricular focused (RVF) view.^29^ This view was found to provide a more accurate measurement of RAV compared to using 3DE as a reference.^29^

Accordingly, we aimed to investigate the relative accuracy of the 2DE-RVF and the 2DE-4Ch apical views to measure RAV in patients with STR, using RAV measured by 3DE as a reference. We have also evaluated the strength of the associations of the RAVs measured using the three echocardiographic modalities with the risk of clinical events

## Methods

### Population and study design

From the database of the FUTURE 3DE study (ClinicalTrials.gov Identifier: NCT05747404) we selected the patients with STR who had the following three echocardiography datasets acquired in the same echocardiographic study: (i) conventional apical 4Ch view including the RA; (ii) apical RVF view, focused on the RA; (iii) multi-beat 3DE dataset of the RA with a temporal resolution higher than 15 vps. Exclusion criteria were: age under 18 years, previous surgical or transcatheter tricuspid valve (TV) repair, and poor image quality. For the purposes of this study, the RAV volumes were all re-measured in a blinded way by an expert operator (M.G.).

Clinical information during follow-up was obtained either via telephone interview or by reviewing electronic medical records of routine outpatient visits and hospital admission. Physicians who were unaware of the patients’ echocardiographic characteristics assigned the clinical events. The primary endpoint was the occurrence of death from any cause and/or hospitalization for heart failure. The present study was approved by the Ethics Committee of the Istituto Auxologico Italiano, IRCCS (record #2020_04_21_06, approved on 21 April 2020).

### 2DE and 3DE image acquisition and analysis

**Figure 1:**
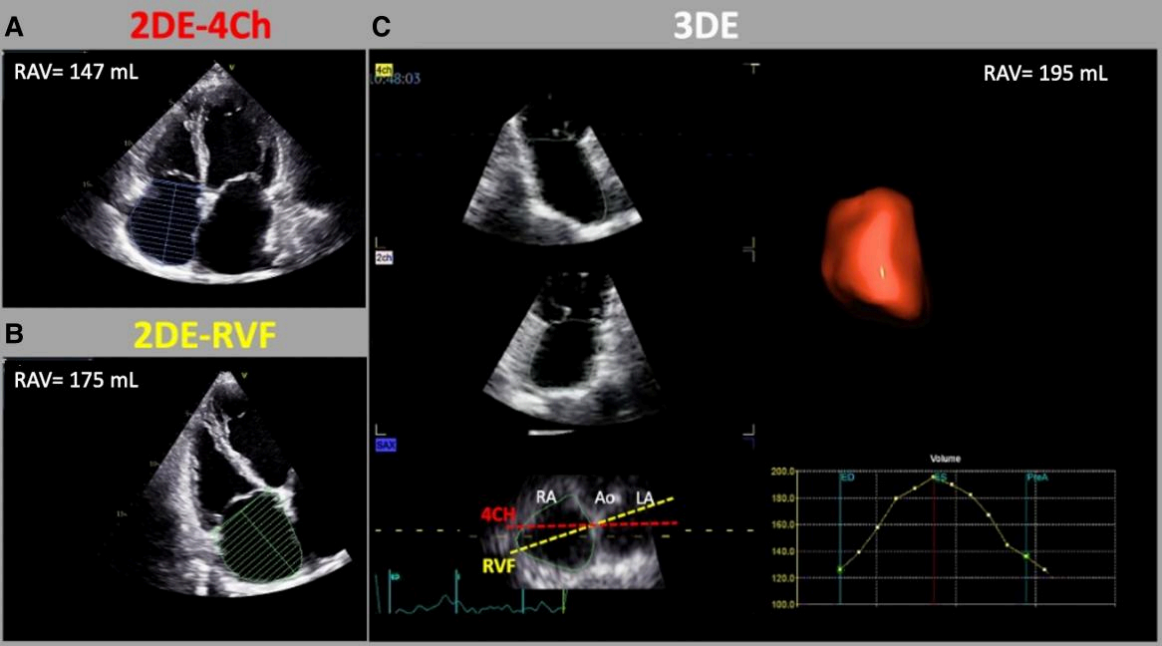
Measurement of RAV by 2DE using apical four-chamber view (*A*), apical right ventricular-focused view (*B*), and 3DE (*C*). 2DE, two-dimensional echocardiography; 3DE, three-dimensional echocardiography; 4Ch, four-chamber; Ao, aortic valve; LA, left atrium; RA, right atrium; RVF, right ventricular focused.

Patients underwent standard 2D and Doppler echocardiography studies using commercially available Vivid E9/E95 (GE HealthCare, UK) equipped with 4 V and 4Vc-D matrix 4D volume phased-array transducers, respectively. Images were analysed offline using EchoPAC 204 by an experienced researcher. 3DE acquisitions of the RV, TV, and RA were obtained using electrocardiogram gating over four to six consecutive cardiac cycles during a single breath-hold.^21^ In patients with atrial fibrillation, multi-beat datasets of two to three consecutive beats (in patients with regular cardiac cycles) or single-beat 3DE dataset were obtained.^33,34^ Conventional and advanced echocardiography parameters were obtained according to the most recent recommendations.^33,35–42^ We used the 4D auto-RVQ software package (EchoPac 204, GE) to measure RV end-diastolic and end-systolic volumes.^32,33^ Atrial and ventricular phenotypes of STR were defined according to the current international consensus.^14,43^ The measurements of the maximum RAV by 2DE were performed at the end systolic frame applying the single-plane area-length algorithm in both the apical 4Ch and the RVF views (*Figure 1*).^35^ The procedure for obtaining an RVF view was as follows: (i) the probe was positioned laterally compared to the conventional 4Ch view and it was directed towards the right shoulder to centre the left ventricular (LV) apex within the scan sector and display the largest RV dimensions; (ii) we took care that neither the aortic valve (too anterior orientation) nor the coronary sinus (too posterior orientation) was displayed within the scan sector and that only the interatrial septum was visible; (iii) finally, the RVF view was further optimized in terms of orientation, depth, and gain, to avoid RA foreshortening and to visualize the entire RA throughout the cardiac cycle.^33,36,37^

3DE RAV was obtained using a commercially available semiautomated software package designed for the left atrium (4D Auto Left Atrium Quantification-LAQ) adapted to the RA^15,16,29,32^ (*Figure 1*). To reduce the effects of the interobserver variability on the comparisons among the RAV obtained using the different echocardiography techniques, the 2DE and 3DE RAV were all re-measured off-line in a blinded way (first she performed all the measurements using the 4CH, then all measurements using the RVF, and finally measured the 3DE datasets) from a single expert operator (M.G.). The reference values reported in the World Alliance Societies of Echocardiography (WASE) study were used.^31^ RA sphericity index was calculated as the ratio of the RAV measured by 3DE to the volume of a hypothetical sphere with the RA length (measured by RVF) as its diameter.^44^ To assess the reproducibility of RAV measurements, a subgroup of 30 patients underwent repeated measurements of RAV by the same operator a second time and then by a second experienced trained operator, blinded to all prior measurements. Intraobserver and interobserver variability were reported as intraclass correlation (ICC).

### Statistical analysis

Statistical analysis was performed using SPSS software, version 28 (SPSS Inc., IBM corp., Chicago, IL, USA). Continuous data were reported as mean ± standard deviation or median ± interquartile range (IQR), after testing for normal distribution by Kolmogorov–Smirnov test. Categorical variables were expressed as percentages. Analysis of variance, Kruskal–Wallis H test, and *χ*^2^ test were used to compare continuous data (with normal and non-normal distribution, respectively) and categorical data, respectively. Bonferroni’s *post-hoc* analysis was applied for comparison between pairs. Differences between variables were considered significant for *P* values <0.05. The absolute (mL/m^2^) and relative (% of the 3D RAV) differences between the measures obtained from the 2D methods (both apical 4Ch vs. RVF-views) and the 3DE were plotted in Bland–Altman analysis. Spearman's rank correlation was used to measure the strength and direction of association between the sphericity index of the RA and the difference in RAV obtained by RVF and 4Ch. The receiver operating characteristic (ROC) curve analysis and the area under the curves (AUC) were used to compare the association of the three methods used to measure RAVs with the composite endpoint.

## Results

### Characteristics of the included patients

The flow chart of the study and the number of excluded patients is shown in Supplementary data online, *Figure S1*. We included 384 patients with mild (131 patients, 34%), moderate (138 patients, 36%), and severe (115 patients, 30%) STR. Of them, 91 (24%) had atrial STR and 293 (76%) had ventricular STR. The demographic, clinical, and echocardiography characteristics of the study population are summarized in *Table 1* and Supplementary data online, *Table S1*.

**Table 1:** Demographic and echocardiographic characteristics of study population

	Whole population	TR mild	TR moderate	TR severe	*P* value
	(*n* = 384)	(*n* = 131)	(*n* = 138)	(*n* = 115)	
Male (*n*, %)	172 (45)	52 (40)	67 (49)	53 (46)	0.326
Age (years)	65 ± 21	56 ± 22	67 ± 20*	72 ± 19*	<0.001
Body surface area (m^2^)	1.75 ± 0.21	1.72 ± 0.19	1.75 ± 0.21	1.80 ± 0.21*	0.011
Heart rhythm					<0.001
Sinus rhythm	273 (71)	113 (86)	97 (70)	63 (55)	
Atrial fibrillation	111 (29)	18 (14)	41 (30)	52 (45)	
Left ventricle ejection fraction (%)	56 ± 12	60 ± 9	55 ± 12*	53 ± 14*	<0.001
Left atrial volume index (mL/m^2^)	44 (31–61)	33 (25–44)	47 (34–60)*	57 (42–73)*, **	<0.001
RVF RAVmax index (mL/m^2^)	45 (30–65)	29 (24–38)	46 (33–60)*	65 (50–81)*, **	<0.001
4Ch RAVmax index (mL/m^2^)	38 (25–59)	25 (19–33)	40 (28–52)*	59 (45–78)*, **	<0.001
3D RAVmax index (mL/m^2^)	50 (36–73)	35 (30–46)	50 (39–71)*	75 (61–92)*, **	<0.001
TR EROA (cm^2^)	0.27 (0.15–0.39)	0.12 (0.07–0.14)	0.26 (0.20–0.30)*	0.43 (0.36–0.53)*, **	<0.001
TAPSE (mm)	20 ± 5	22 ± 5	20 ± 5*	18 ± 5*, **	<0.001
RV free wall longitudinal strain (%)	22 ± 7	25 ± 6	22 ± 7*	19 ± 6*, **	<0.001
3D RV end diastolic volume index (mL/m^2^)	86 ± 35	75 ± 30	84 ± 34	102 ± 38*, **	<0.001
3D RV ejection fraction (%)	50 ± 11	52 ± 10	50 ± 11	47 ± 11*, **	<0.001
Pulmonary arterial systolic pressure (mm Hg)	39 ± 18	33 ± 15	39 ± 16*	46 ± 21*, **	<0.001

All continuous variables were expressed as mean ± SD or median and IQR. All discrete variables were expressed as absolute number and percentage.

3D, three-dimensional; 4Ch, four-chamber view; EROA, effective regurgitant orifice area; LAV, left atrial volume; LV, left ventricle; RA, right atrium; RAV, right atrial volume; RVF, right ventricular focuses; RV, right ventricle; TAPSE, tricuspid annulus plane systolic excursion.

^*^ *P* < 0.05 vs. mild STR with Bonferroni’s *post*-*hoc* analysis.

^**^ *P* < 0.05 vs. moderate STR with Bonferroni’s *post-hoc* analysis.

### Agreement between 2DE and 3DE for RAV measures and reproducibility

**Figure 2:**
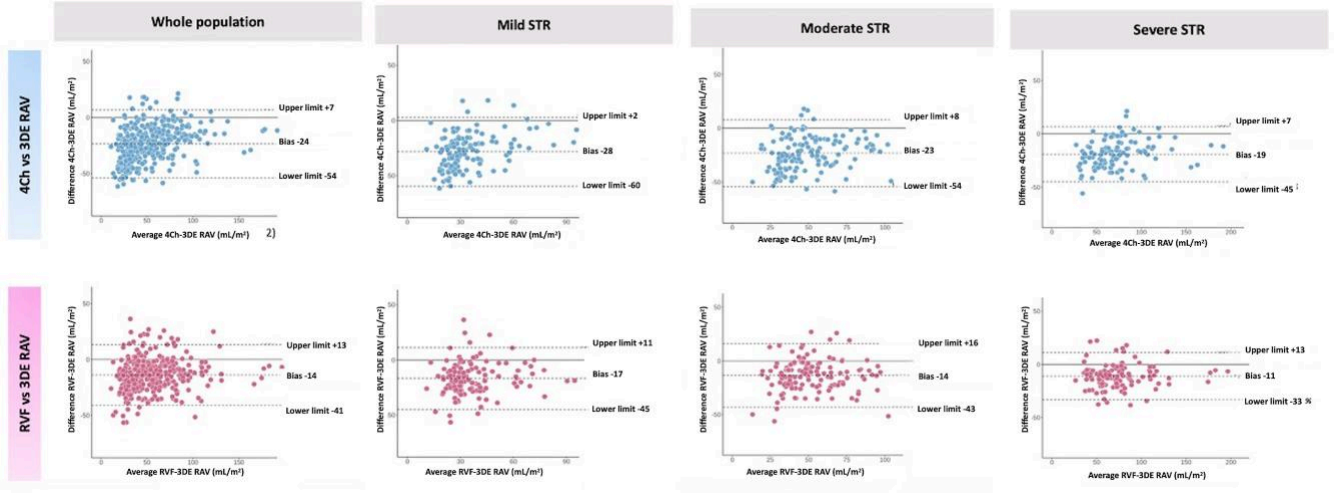
Bland–Altman plots show the relative biases for the agreement between the RAVs calculated from the two-dimensional apical four-chamber and right ventricular focused views with those measured by 3DE in the whole population and the groups of patients with different grades of STR. 3DE, three-dimensional echocardiography; 4Ch, four-chamber; RAV, right atrial volume; RVF, right ventricular focused; STR, secondary tricuspid regurgitation.

RAV significantly increased with the worsening of the STR severity, irrespective of the measurement method used (4Ch, RVF, or 3DE) (*Table 1*). Considering the whole study population, RAVs were significantly larger when measured using 3DE (50 mL/m^2^, IQR: 36–73) in comparison to RVF (45 mL/m^2^, IQR: 30–65) and 4Ch (38 mL/m^2^, IQR: 25–59) (*P* < 0.001 for both differences) (see Supplementary data online, *Figure S2*). The Bland–Altman plots showed that the RAV calculated using the 4Ch view provided a 24% underestimation of the 3DE RAV, significantly larger than the 14% underestimation when measurements were obtained on RVF (*P* < 0.001) (*Figure 2* and Supplementary data online, *Table S2*). The difference in the extent of the underestimation of the RAV remained significant for each degree of STR (*P* value <0.001 for all the comparisons) (see Supplementary data online, *Table S3*). As the severity of STR increased, the underestimation of RAV by 4Ch and RVF, compared with 3DE, decreased (4Ch: −28% in mild STR vs. −23% in moderate STR vs. −19% in severe STR, *P* = 0.001; and RVF: −17% in mild STR vs. −14% in moderate STR vs. −11% in severe STR, respectively, *P* = 0.005) (*Figure 2*). Additionally, ICC for the agreement with 3DE improved for both 4Ch and RVF with the increase in TR grade (see Supplementary data online, *Table S3*). The difference in the underestimation of RAV by 4Ch vs. RVF was inversely related to RA sphericity (R Spearman: −0.2, *P* = 0.001).

**Figure 3:**
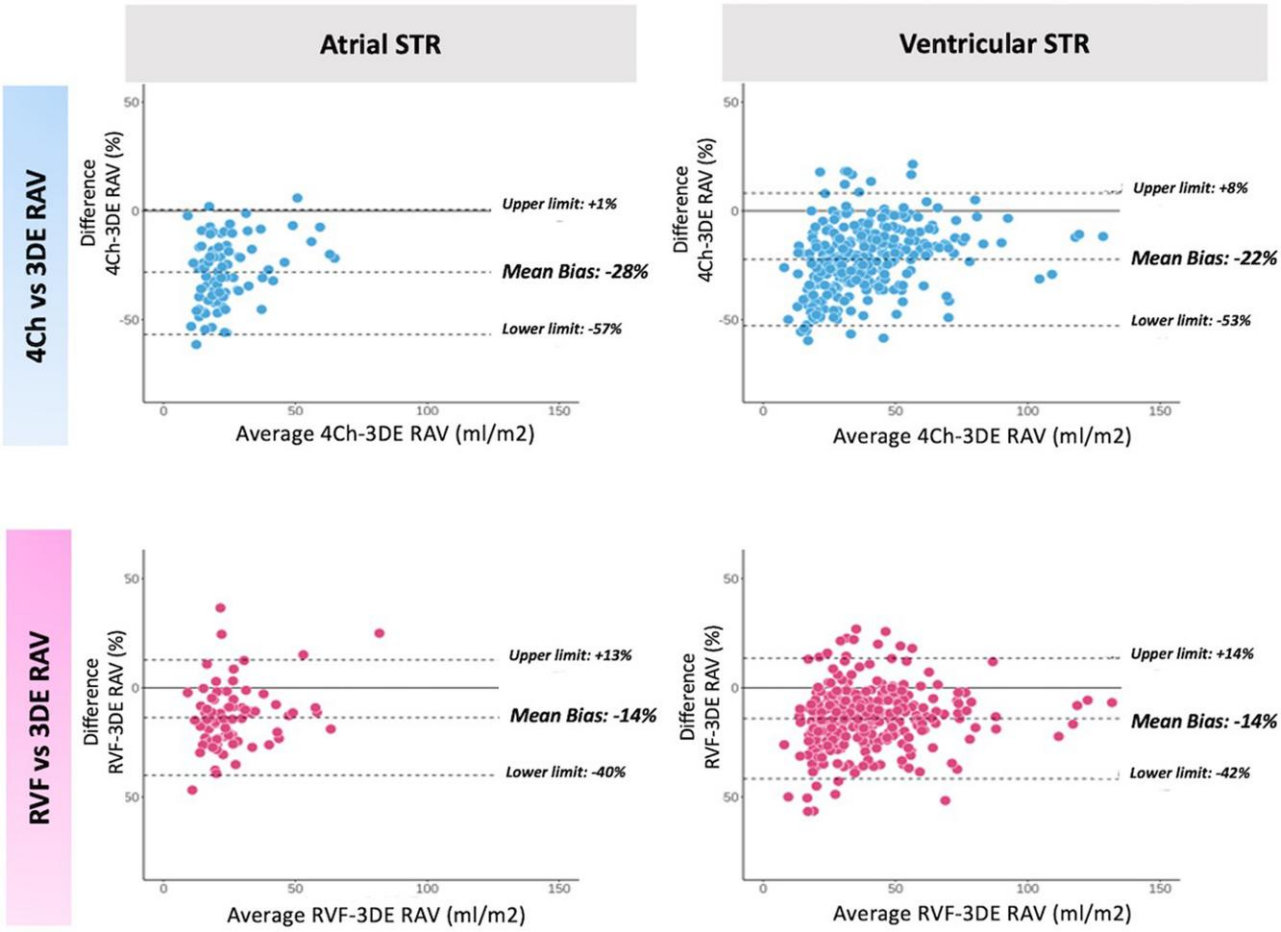
Bland–Altman with a relative bias for the agreement between 2DE and 3DE methods for RAV measure in patients with atrial and ventricular STR. 3DE, three-dimensional echocardiography; 4Ch, four-chamber; RAV, right atrial volume; RVF, right ventricular focused; STR, secondary tricuspid regurgitation.

RAV was underestimated in both the atrial and the ventricular STR phenotypes when the 4Ch view was used to obtain measurements (4Ch vs. RVF: −28 vs. −13%, *P* < 0.001 in atrial STR; −22 vs. −14% in ventricular STR, *P* < 0.001) (see Supplementary data online, *Table S4* and *Figure 3*). Notably, when 4Ch was used, the underestimation of RAV was significantly larger in atrial compared to ventricular STR phenotype (−28 vs. −22%, *P* = 0.002), across all the STR severities (4Ch in severe STR: −24% in atrial STR vs. −18% in ventricular STR, *P* = 0.043; 4Ch in moderate STR: −27 vs. −22%, *P* = 0.041; 4Ch in mild STR −29 vs. −27%, *P* = 0.049); using RVF (−12 vs.—11%, *P* = 0.381 in severe STR, −13 vs. −12%, *P* = 0.341 in moderate STR; −18 vs. −16%, *P* = 0.178 in mild STR). Conversely, when the RVF view was used, the extent of underestimation of 2DE vs. 3DE RAV was similar in both the atrial and ventricular STR phenotypes (see Supplementary data online, *Figure S2*).

Intraobserver and interobserver variability were higher when RAVs were obtained from the 4Ch compared to the RVF and 3DE (see Supplementary data online, *Table S5*).

### Comparison of RA volumes obtained by the different techniques in detecting RA dilation and their association with clinical outcome

Compared with WASE reference values,^42^ 243 patients (63%) had a dilated RAV when using the 4Ch, 276 (72%) when using the RVF, and 300 (78%) when using the 3DE. (*P* = 0.03 for the difference). These results indicate that the 4Ch has lower sensitivity (80 vs. 89%; *P* = 0.019) and higher specificity (96 vs. 88%; *P* = 0.001) than RVF in detecting RA dilation (see Supplementary data online, *Table S6*).

**Figure 4:**
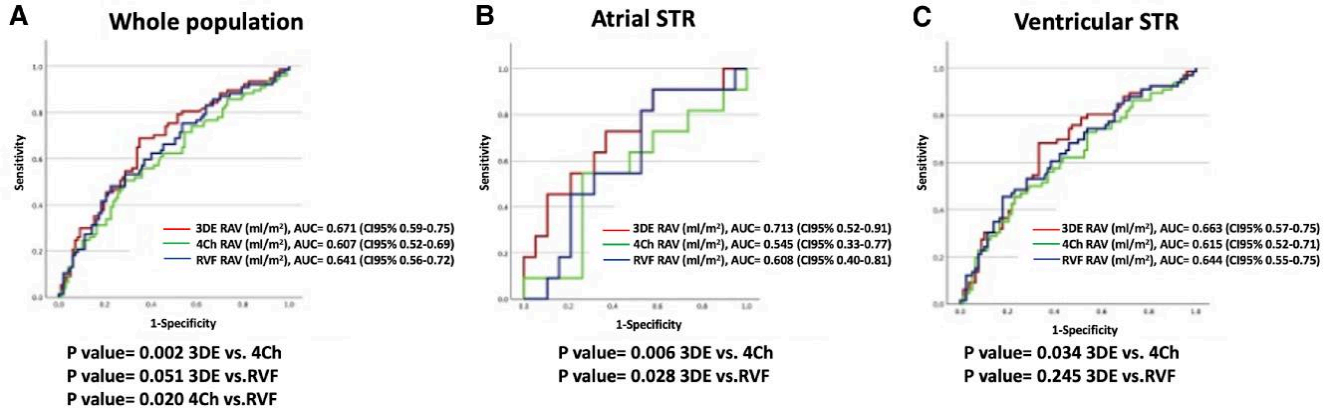
ROC curve analysis to assess the association of the measures of RAV by using apical four-chamber apical view, apical right ventricular-focused view, and 3DE with the composite endpoint in the whole population (*A*), in patients with atrial (*B*), and ventricular (*C*) STR. 3DE, three-dimensional echocardiography; 4Ch, four-chamber; RAV, right atrial volume; RVF, right ventricular focused; STR, secondary tricuspid regurgitation.

One hundred eighty-four patients with clinical follow-up (mean 431 ± 365 days) were included in the analysis for association with outcomes. At ROC analysis, 3DE RAV showed the best association with outcome (AUC 0.67, 95% CI 0.59–0.75), followed by 2DE RAV on RVF (AUC 0.64, 95% CI 0.56–0.72, *P* = 0.051 vs. 3DE RAV), and 2DE RAV on 4Ch (AUC 0.61, 95% CI 0.52–0.69, *P* = 0.02 vs. 2DE RAV on RVF and *P* = 0.021 vs. RAV by 3DE) (*Figure 4A*). Similarly, in atrial STR, the AUC of the 3DE RAV was significantly larger than those of the 2DE RAVs obtained from the 4Ch (*P* = 0.001) and the RVF (*P* = 0.028). Conversely, in ventricular STR, the AUC of the 2DE RAV by RVF and 3DE RAV were similar (*Figure 4B* and *C*).

## Discussion

The results of the present study may be summarized as following: (i) in patients with STR, RAVs measured by 2DE, either using the 4Ch or the RVF, are systematically smaller than those obtained by 3DE; (ii) the extent of the underestimation of 3DE RAVs is significantly larger using the 4Ch than the RVF view, especially at lower grade of STR, and in the atrial than in the ventricular STR; (iii) the association with outcomes is the highest for RAV measured by 3DE, followed by RVF, and last by 4Ch.

Although it is becoming increasingly evident that 3DE allows more accurate measurement of cardiac chamber volumes, this technique has not been widely adopted to assess right heart chambers and RA in particular.^30^ Current guidelines recommend that an apical 4Ch view focused on RA should be used to obtain RAV.^35,36^ However, recent evidence showed that this approach systematically underestimates RAV.^28,29,31^ We have previously reported that, in unselected patients referred for clinically indicated echocardiography, taking measurements on an RVF instead of the conventional 4Ch view yielded more accurate RAV measurements compared to cardiac magnetic resonance and 3DE.^29^ The present study is the first one that focuses only on patients with STR to explore the accuracy and the reproducibility of the RAVs obtained from 3DE vs. RVF and 4Ch; furthermore, the present is the first study exploring the association with clinical outcomes of the measurements obtained by these different methods.

First, our study confirmed that 2DE RAV underestimates RAVS measured by 3DE, irrespective of the 2D view used to take the measurements. Secondly, using the RVF, we obtained larger RAVs than those obtained from the conventional 4Ch view. Around 21% of the patients were reclassified as having RA dilation when measurements were taken on the RVF compared with 4Ch.

Interestingly, the RAV underestimation by 2DE vs. 3DE and of 4Ch vs. RVF was larger in patients with less severe STR, as well as in patients with the atrial STR compared to the ventricular STR phenotype. These results confirm the importance of using 3DE or, at least the 2DE RVF, to measure RAV in the first phases of RA remodelling and the atrial phenotype. Finally, we found that a more asymmetrical shape (i.e. lower sphericity index) of the RA was correlated to a larger difference between the RAVs obtained from RVF and 4Ch (see Supplementary data online, *Figure S2*).

Thirdly, we have also explored the association of RAVs obtained by the different echocardiographic techniques with outcomes. Interestingly, the RAVs measured by 3DE showed the closest association with clinical outcomes. The RAV obtained by RVF had similar clinical performance to 3DE, while RAVs measured by 4Ch showed a significantly weaker association with events.

Finally, measurements performed by 3DE and RVF yielded a higher reproducibility than measurements obtained from the conventional 4Ch.

### Clinical implications

Accurate quantification of the RA size is essential to precisely phenotyping patients with STR.^14,45–47^ and to distinguish patients with the atrial from the ventricular STR phenotype, with the former exhibiting a reportedly better prognosis following conservative treatment or repair procedure.^23,24^ Furthermore, accurate and reproducible identification of RA remodelling would help in the clinical follow-up of patients with moderate or severe STR, providing insights into the hemodynamic relevance of the STR and its progression.^13,47^ Finally, accurate assessment of RA volume and function, in association with left atrial size and function evaluation, allows for stratifying the risk of recurrence of atrial fibrillation after elective electrical cardioversion and facilitates tailored management based on individual patients’ characteristics.^8,22^

### Limitations

Our results should be confirmed in prospective multi-centre studies enrolling larger cohorts of patients with STR. Currently, there is no commercially available dedicated software package to measure RA volumes using 3DE. To overcome this, we adapted a software package originally developed to measure the left atrium. However, this software package has been reported to be accurate in measuring the RAV using 3DE.^28,29^

Since in our daily clinical practice, we routinely use the RVF to obtain RAV and do not store the conventional 4Ch optimized for RAV assessment, the percentage of patients excluded from the present analysis was high. Even if the ROC analysis does not account for other confounders, the present results should be considered as proof of concept to raise awareness of the clinical implication of the underestimation of RAV.

## Conclusions

In patients with STR, the use of the 2DE 4Ch for assessing RAV systematically underestimates the RAV obtained by 3DE. However, this underestimation can be significantly reduced using a dedicated 2DE RVF view to measure the RAV. For laboratories without 3DE capabilities or expertise, measuring RAV from dedicated RVF views will provide a more accurate assessment of RA size.

## Supplementary data

Supplementary data are available at *European Heart Journal-Cardiovascular Imaging* online.

## Supplementary Material

jeae186_Supplementary_Data

## Data Availability

The data underlying this article will be shared on reasonable request to the corresponding author.
