# Supplementary material for: Assessing right atrial size in patients with tricuspid regurgitation: importance of the right ventricular-focused view
Source: Eur Heart J Cardiovasc Imaging. 2024 Jul 25;25(12):1743–50. doi: 10.1093/ehjci/jeae186 (PMC11601883; doi:10.1093/ehjci/jeae186)
Supplement: jeae186_Supplementary_Data [file jeae186_supplementary_data.zip › jeae186_Supplementary_Data.docx]

**Table S1:** Demographic and echocardiographic characteristics of study population divided into the atrial and ventricular phenotype

|  | Atrial STR  (n = 91) | Ventricular STR  (n = 293) | p value |
| --- | --- | --- | --- |
| Male | 44 (48) | 129 (44) | 0.236 |
| Age (years) | 54 ± 23 | 72 ± 16 | <0.001 |
| Body surface area (m^2^) | 1.76 ± 0.19 | 1.75 ± 0.21 | 0.594 |
| Heart rate (bmp) | 70 ± 14 | 75 ± 16 | 0.009 |
| Systolic blood pressure (mmHg) | 127 ± 17 | 127 ± 23 | 0.847 |
| Diastolic blood pressure (mmHg) | 74 ± 11 | 74 ± 12 | 0.615 |
| Heart rhythm at the time of TTE |  |  | <0.001 |
| Sinus rhythm | 27 (30) | 199 (68) |  |
| Atrial fibrillation | 64(70) | 94 (32) |  |
| Mitral regurgitation ≥ moderate | 0 (0) | 58 (20) | <0.001 |
| Left ventricle ejection fraction (%) | 62 ± 5 | 52 ± 14 | <0.001 |
| LAV index (mL/m^2^) | 32 (25-43) | 53 (41-71) | <0.001 |
| RVF RAVmax index (mL/m^2^) | 32 (25-45) | 53 (38-71) | <0.001 |
| 4Ch RAVmax index (mL/m^2^) | 26 (21-39) | 49 (34-67) | <0.001 |
| 3D RAVmax index (mL/m^2^) | 40 (31-52) | 63 (45-81) | <0.001 |
| 2D RA longitudinal strain (%) | 34 (17-44) | 12 (8-21) | <0.001 |
| 3D-RAEF (%) |  |  |  |
| 2D TR EROA (cm^2^) | 0.20 ±0.10 | 0.30 ±0.20 | 0.019 |
| Reg Volume (mL) | 16 ± 8 | 27 ± 15 | 0.002 |
| Tethering height (mm) | 9 ± 4 | 16 ± 8 | 0.045 |
| TAPSE (mm) | 24 ± 4 | 19 ± 4 | <0.001 |
| RV- End diastolic area (cm^2^) | 24 ± 9 | 26 ± 10 | 0.015 |
| RV- End systolic area (cm^2^) | 13 ± 6 | 17 ± 8 | <0.001 |
| RV-Fractional area shortening (RV-FAC) | 40 ± 6 | 34 ± 8 | 0.003 |
| RV Free Wall Strain (%) | 26 ± 5 | 20 ± 6 | <0.001 |
| RV global longitudinal strain (%) | 21 ± 4 | 17 ± 5 | <0.001 |
| 3D RV end diastolic volume index (mL/m^2^) | 60 ± 10 | 93 ± 37 | <0.001 |
| 3D RV end systolic volume index (mL/m^2^) | 25 ± 5 | 50 ± 28 | <0.001 |
| 3D RV ejection fraction (%) | 59± 6 | 47 ± 10 | <0.001 |
| Pulmonary artery systolic pressure (mm Hg) | 26 ± 5 | 43 ± 19 | <0.001 |

All continuous variables were expressed as mean ± SD or median and interquartile range. All discrete variables were expressed as absolute number and percentage. *The p values depict differences between grades TR and are calculated by analysis of variance (ANOVA) and Kruskal-Wallis H test for continuous data (with normal and non-normal distribution, respectively), and by chi-square test for categorical data. †p < 0.05 vs. TR mild with Bonferroni’s post hoc analysis. ‡p < 0.05 vs. TR moderate with Bonferroni’s post hoc analysis.

Abbreviations: 4CH: four chamber view; EROA, effective regurgitant orifice area; LAV, left atrial volume; LV, left ventricle; RA, right atrium; RAV, right atrial volume; RegVol, regurgitant volume ; RVF, right ventricular focuses; RV, right ventricle; TAPSE: tricuspid annulus plane systolic excursion.

**Table S2:** Absolute (ml/m2) and relative (%) bias with limits of agreement (LOA) and intraclass correlation coefficient (ICC) obtained comparing RAVs max measured with 2DE by using the conventional apical 4Ch view (upper part) and the RVF-view (lower part) in the whole population (n=384 patients).

| Correlations between 2DE-4Ch view and 3DE | | | |
| --- | --- | --- | --- |
|  | Bias | LOA | ICC |
| RAV (mL/m^2^) | -13 | 20 | 0.848 |
|  | (-24%)* | (±31%) |  |
| Correlations between 2DE-RVF view and 3DE | | | |
|  | Bias | LOA | ICC |
| RAV (mL/m^2^) | -8 | 19 | 0.906 |
|  | (-14%) | (±27%) |  |

Abbreviations: 4CH: four chamber view; 2D: two dimensional, 3D: three dimensional; ; ICC: Intraclass correlation coefficiency; LOA: limits of agreement; RAV: right atrial volume.

***P <0.001 for comparison between bias with 2DE-4Ch vs 2DE-RVF**

**Table S3:**  Absolute (ml/m2) and relative (%) bias with limits of agreement (LOA) and intraclass correlation coefficient (ICC) obtained comparing RAVs max measured with 2DE by using the conventional apical 4Ch view (upper part) and the RVF-view (lower part) in patients grouped on the base of STR severity.

**Correlations between 2DE-4Ch view and 3DE**

|  | | | | | | | | | | |
| --- | --- | --- | --- | --- | --- | --- | --- | --- | --- | --- |
|  | STR mild | | | STR moderate | | | STR severe | | | |
|  | Bias | LOA | ICC | Bias | LOA | ICC | Bias | LOA | ICC |  |
| **RAV (mL/m^2^)** | -11*^£^  (-28%) | 14  (±31%) | 0.742 | -13*^£^  (-23%) | 21  (±31%) | 0.781 | -15*^£^  (-19%) | 24  (±26%) | 0.827 |  |
| **Correlations between 2DE-RVF view and 3DE** | | | | | | | | | | |
|  | STR mild | | | STR moderate | | | STR severe | | | |
|  | Bias | LOA | ICC | Bias | LOA | ICC | Bias | LOA | ICC |  |
| **RAV (mL/m^2^)** | -7**  (-17%) | 13  (±28%) | 0.834 | -8**  (-14%) | 23  (±30%) | 0.826 | -9**  (-11%) | 19  (±22%) | 0.910 |  |

Abbreviations: 4CH: four chamber view; 2D: two dimensional, 3D: three dimensional; ; ICC: Intraclass correlation coefficiency; LOA: limits of agreement; RAV: right atrial volume; STR: secondary tricuspid regurgitation

*p value=0.001 for comparison of bias between mild and moderate, moderate and severe using 2DE-4Ch;

** p value =0.005 for comparison of bias between mild and moderate, moderate and severe using 2DE-RVF

£ = P <0.001 for comparison between bias with 2DE-4Ch vs 2DE-RVF

**Table S4:** Absolute (ml/m2) and relative (%) bias with limits of agreement (LOA) and intraclass correlation coefficient (ICC) obtained comparing RAVs max measured with 2DE by using the conventional apical 4Ch view (upper part) and the RVF-view (lower part) in patients grouped on the base of STR phenotype (atrial vs ventricular).

| Correlations between 2DE-4CH view and 3DE | | | | | | |
| --- | --- | --- | --- | --- | --- | --- |
|  | Atrial STR (n=91) | | | Ventricular STR (n=293) | | |
|  | Bias | LOA | ICC | Bias | LOA | ICC |
| **RAV (mL/m^2^)** | -9  (-28%)* ^£^ | 14  (±27%) | 0.872 | -13  (-22%)^£^ | 21  (±22%) | 0.839 |
| **CORRELATIONS BETWEEN 2DE-RVF VIEW AND 3DE** | | | | | | |
|  | Atrial STR (n=91) | | | ventricular STR (n=293) | | |
|  | Bias | LOA | ICC | Bias | LOA | ICC |
| **RAV (mL/m^2^)** | -6  (-13%)** | 12  (±14%) | 0.950 | -11  (-14%) | 19  (±14%) | 0.945 |

Abbreviations: 4CH: four chamber view; 2D: two dimensional, 3D: three dimensional; ; ICC: Intraclass correlation coefficiency; LOA: limits of agreement; RAV: right atrial volume; STR: secondary tricuspid regurgitation

*p value=0.002 for comparison with bias with 2D-4Ch in ventricular STR; ** p value < 0.001 for comparison with bias using 2DE-4Ch view; £ P <0.001 for comparison between bias with 2DE-4Ch vs 2DE-RVF

**Table S5:** Reproducibility of right atrial volumes obtained with the echocardiographic methods.

| RAV (ml/m2) | Intraclass correlation coefficiency (95% CI) for interobserver variability | Intraclass correlation coefficiency (95% CI) for intraobserver variability |
| --- | --- | --- |
| 2DE-4Ch | 0.894(0.691-0.957) | 0.889(0.669-0.955) |
| 2DE-RVF | 0.945(0.880-0.872) | 0.955(0.904-0.979) |
| 3DE | 0.972(0.943-0.987) | 0.982(0.964-0.992) |

Abbreviations: 4CH: four chamber view; 2D: two dimensional, 3D: three dimensional; CI: Confidence Interval; RAV: right atrial volume; STR: secondary tricuspid regurgitation

**Table S6.**  Classification of RAV into normal or dilated according to the three methods.

|  | 3D RAVmax index (mL/m2) | | RVF RAVmax index (mL/m2) | |
| --- | --- | --- | --- | --- |
|  | Normal | Dilated | Normal | Dilated |
| Men | 45 | 167 | 49 | 163 |
| Women | 39 | 133 | 59 | 113 |

Abbreviations. 3D: three dimensional, 4ch: four chamber view, AUC: area under the curve; CI: confidence interval; RAV: right atrial volume. RVF: right ventricular focused view.
