# Supplementary figures and images for: Assessing right atrial size in patients with tricuspid regurgitation: importance of the right ventricular-focused view
Source: Eur Heart J Cardiovasc Imaging. 2024 Jul 25;25(12):1743–50. doi: 10.1093/ehjci/jeae186 (PMC11601883; doi:10.1093/ehjci/jeae186)

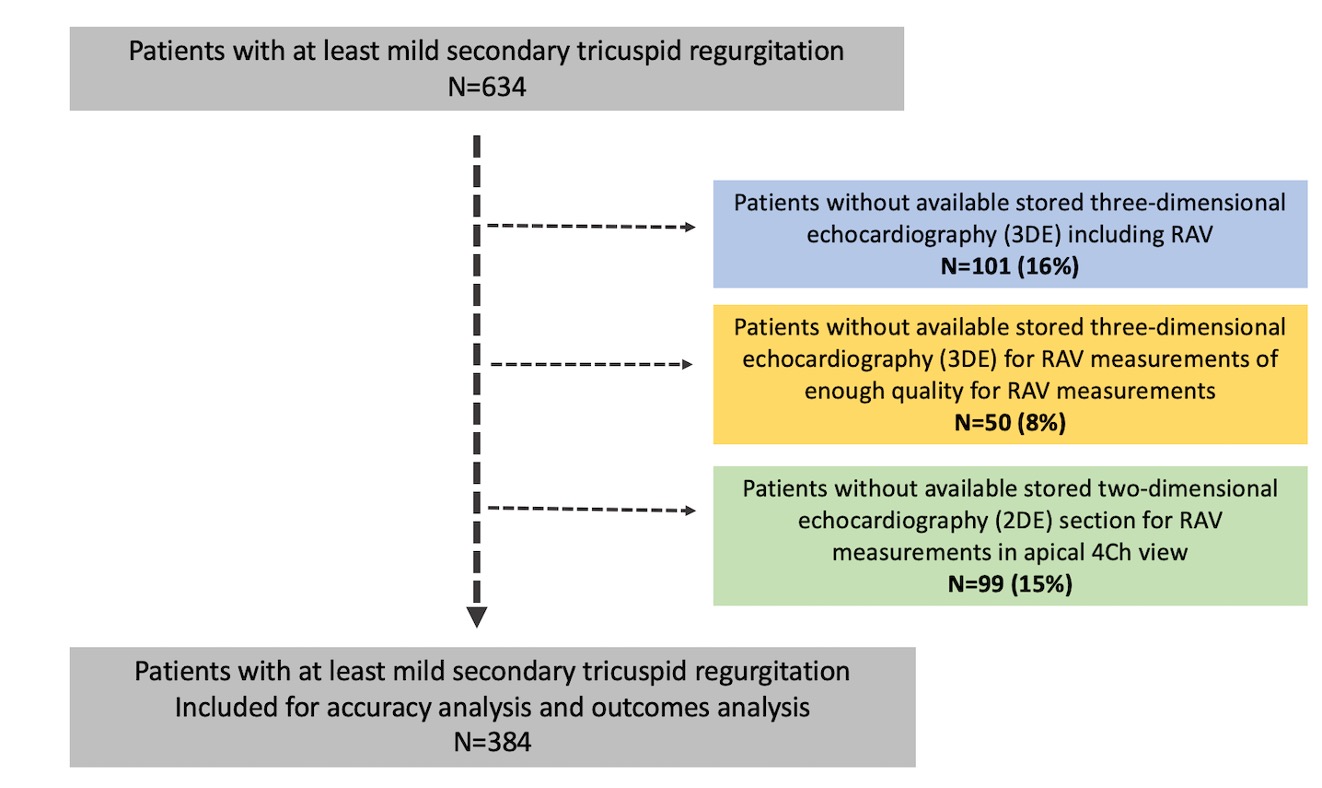

Supplement: jeae186_Supplementary_Data [file jeae186_supplementary_data.zip › FIGURE_S1.jpg]

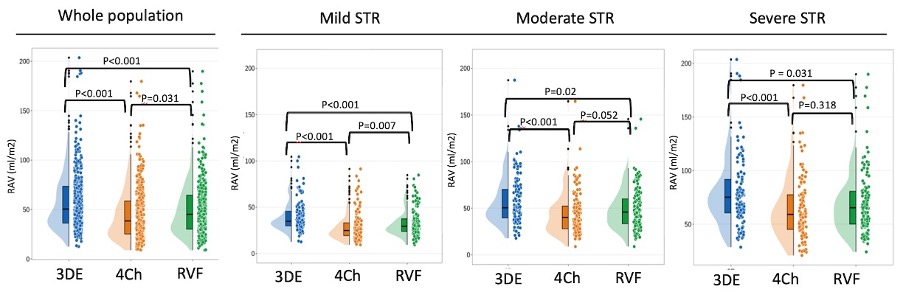

Supplement: jeae186_Supplementary_Data [file jeae186_supplementary_data.zip › FIGURE_S2.jpg]

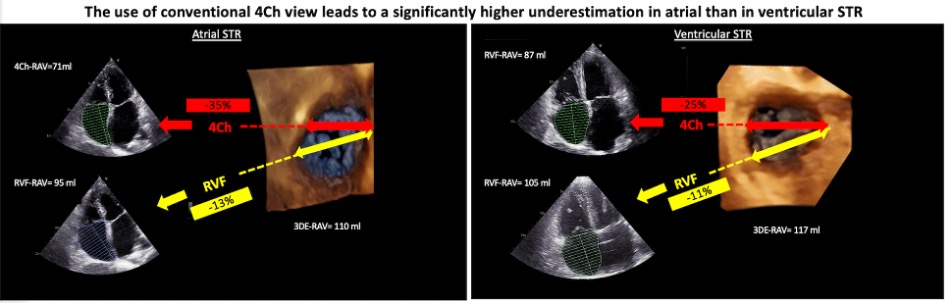

Supplement: jeae186_Supplementary_Data [file jeae186_supplementary_data.zip › FIGURE_S3.jpg]
